# Supplementary material for: Synergistic Adsorption-Catalytic Sites TiN/Ta2O5 with Multidimensional Carbon Structure to Enable High-Performance Li-S Batteries
Source: Nanomaterials (Basel). 2021 Oct 28;11(11):2882. doi: 10.3390/nano11112882 (PMC8623583; doi:10.3390/nano11112882)
Supplement: Supplementary file 1 [file nanomaterials-11-02882-s001.zip › nanomaterials-1404979-supplementary.pdf]

nanomaterials

MDPI

Supplementary Materials:

Synergistic Adsorption–Catalytic Sites TiN/Ta_2O_5 with Multidimensional Carbon Structure to Enable High-Performance Li–S Batteries

Chong Wang [1], Jian-Hao Lu [2], Zi-Long Wang [2], An-Bang Wang [2], Hao Zhang [2], Wei-Kun Wang [2,*], Zhao-Qing Jin [2,*] and Li-Zhen Fan [1,*]

1 Beijing Advanced Innovation Center for Materials Genome Engineering, Institute of Advanced Materials and Technology, University of Science and Technology Beijing, Beijing 100083, China; wangchong18810@163.com

2 Military Power Sources Research and Development Center, Research Institute of Chemical Defense, Beijing 100191, China; hahalujianhao@163.com (J.-H.L.); wangzilong0709@163.com (Z.-L.W.); wab_wang2000@163.com (A.-B.W.); dr.h.zhang@hotmail.com (H.Z.)

* Correspondence: fanlizhen@ustb.edu.cn (L.-Z.F.); wangweikun2002@163.com (W.-K.W.); jinzhaoqing1001@gmail.com (Z.-Q.J.)

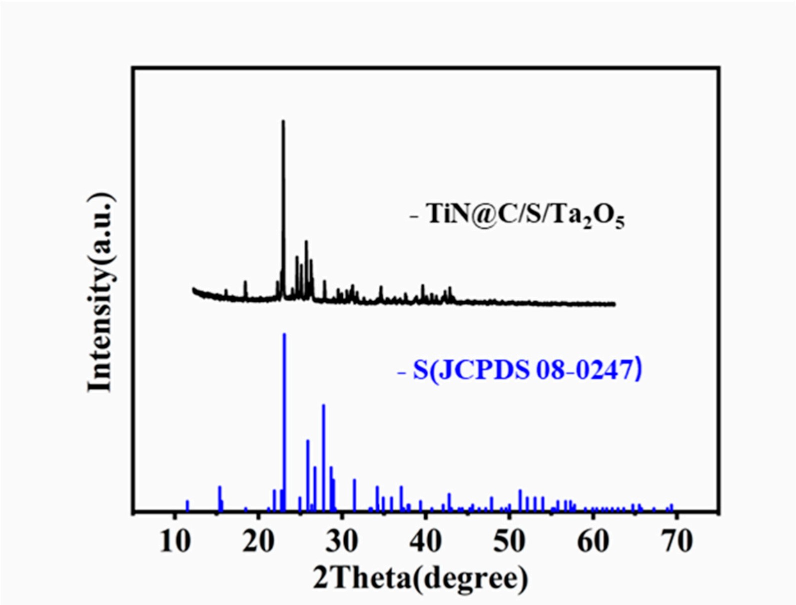

Figure S1. XRD pattern of pure sulfur powder and TiN@C/S/Ta_2O_5 composite.

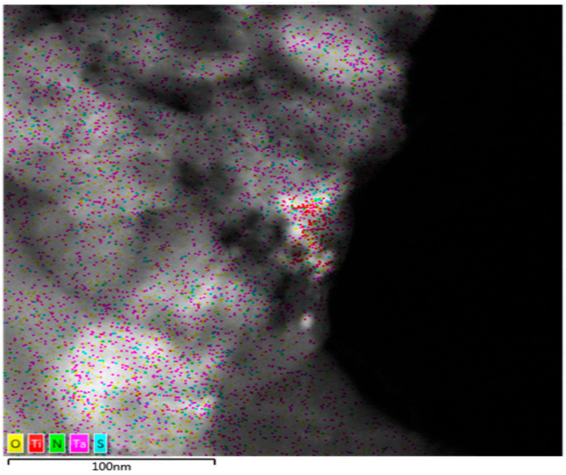

Figure S2. EDS elemental mapping images of TiN@C/S/Ta_2O_5 material.

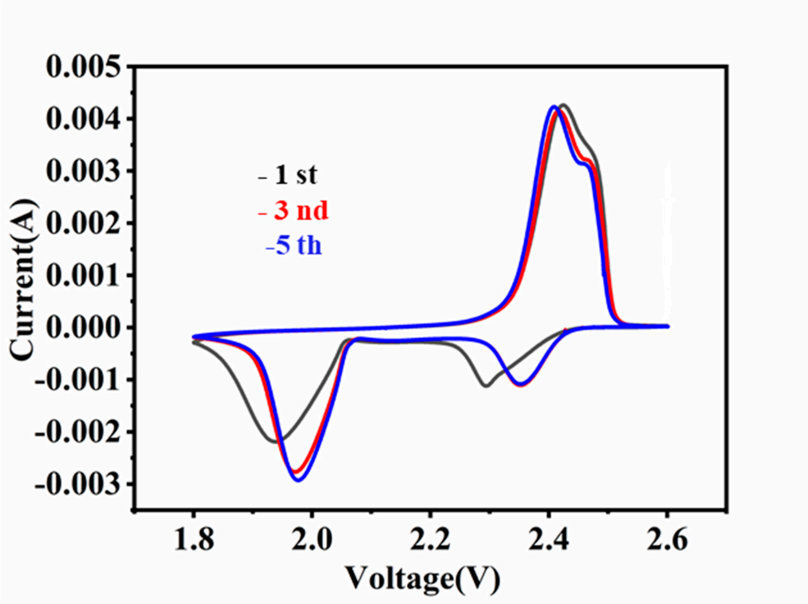

Figure S3. CV profiles of TiN@C/S/Ta_2O_5 at a scan rate of 0.1 mV s^{-1}.

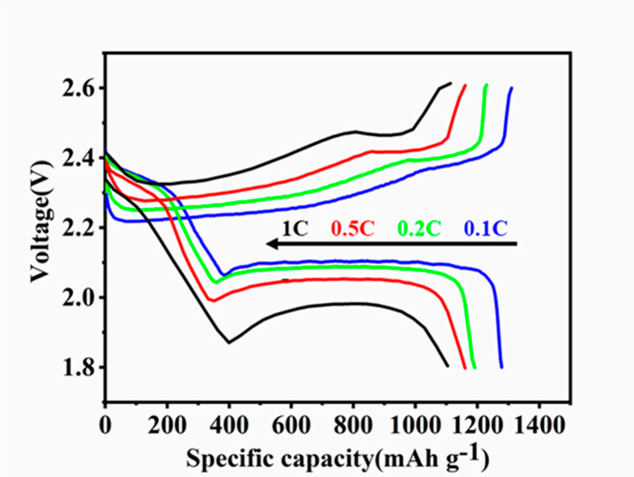

Figure S4. Multi-rate discharge-charge profiles of TiN@C/S/Ta_2O_5.

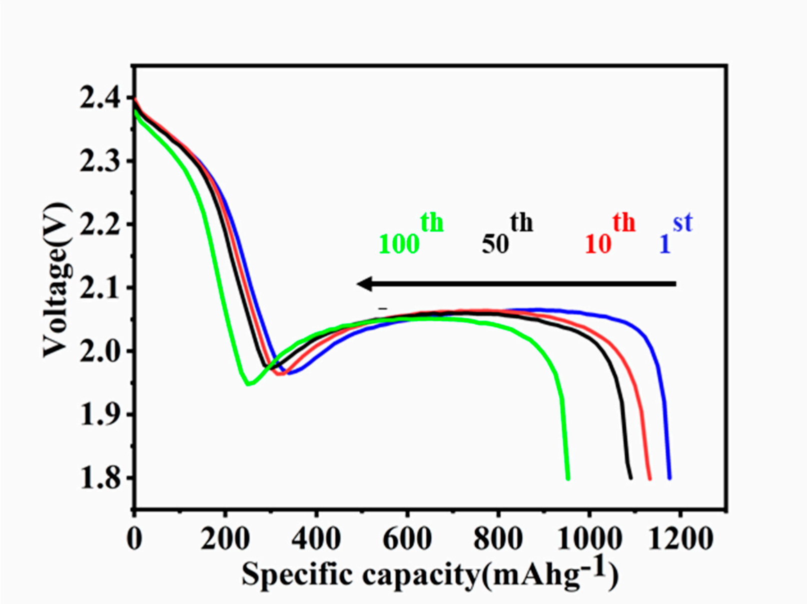

Figure S5. Discharge performance of TiN@C/S/Ta_2O_5 at different cycles at a rate of 0.5 C.

Table S1. Performance Comparison with other sulfur electrodes.

Materials	Cycle Number	Rate C	Capacity Fading Rate	Reference
TiN/Ta_2O_5	300	0.5C	0.192%	This work
$Na_2Fe[Fe(CN)_6]$@PEDOT/S	100	2C	0.15%	[1]
$Ti_3C_2T_x$@Meso-C/S	300	0.5C	0.14%	[2]
$ZnCo_2O_4$@N-RGO/S	200	0.5C	0.2%	[3]

Table S2. Comparison of the pouch cell performance of our work with previously reported work focusing on sulfur cathodes.

Areal S Loadin,g, mg cm^{-2}	E/S Ratio μLmg^{-1}	Cycle Number	Areal Capacity mAhcm^{-2}	Capacity Retention %	Reference
5.3	3.3	50th	5.8	61.63	This work
5.0	7	50th	3.65	82	[4]
5.0	7	50th	4.0	70.26	[5]
4.34	5	80th	2.65	57.3	[6]
4.0	7	30th	2.9	72.03	[7]

References

1. Su, D.; Cortie, M.; Fan, H.; Wang, G. Prussian Blue Nanocubes with an Open Framework Structure Coated with PEDOT as High-Capacity Cathodes for Lithium–Sulfur Batteries. *Adv. Mater.* **2017**, *29*, 1700587.
2. Bao, W.; Su, D.; Zhang, W.; Guo, X.; Wang, G. 3D Metal Carbide@Mesoporous Carbon Hybrid Architecture as a New Polysulfide Reservoir for Lithium-Sulfur Batteries. *Adv. Funct. Mater.* **2016**, *26*, 8746–8756.
3. Sun, Q.; Xi, B.; Li, J.-Y.; Mao, H.; Ma, X.; Liang, J.; Feng, J.; Xiong, S. Nitrogen-Doped Graphene-Supported Mixed Transition-Metal Oxide Porous Particles to Confine Polysulfides for Lithium–Sulfur Batteries. *Adv. Energy Mater.* **2018**, *8*, 1800595.
4. Luo, L.; Li, J.; Asl, H.Y.; Manthiram, A. *In-Situ* Assembled VS4 as a Polysulfide Mediator for High-Loading Lithium–Sulfur Batteries. *ACS Energy Lett.* **2020**, *5*, 1177–1185.
5. Luo, L.; Chung, S.H.; Asl, H.Y.; Manthiram, A. Long-Life Lithium–Sulfur Batteries with a Bifunctional Cathode Substrate Configured with Boron Carbide Nanowires. *Adv. Mater.* **2018**, *30*, 1804149.
6. Kim, M.S.; Kim, M.S.; Do, V.D.; Xia, Y.Y.; Kim, W.; Cho, W. Facile and scalable fabrication of high-energy-density sulfur cathodes for pragmatic lithium-sulfur batteries. *J. Power Sources* **2019**, *422*, 104–112.
7. Zhang, J.; You, C.; Wang, J.; Xu, H.; Zhu, C.; Guo, S.; Zhang, W.; Yang, R.; Xu, Y. Confinement of sulfur species into heteroatom-doped, porous carbon container for high areal capacity cathode. *Chem. Eng. J.* **2019**, *368*, 340–349.
